# Supplementary material for: Search for Indexes to Evaluate Trends in Antibiotic Use in the Sub-Prefectural Regions Using the National Database of Health Insurance Claims and Specific Health Checkups of Japan
Source: Antibiotics (Basel). 2022 Jun 2;11(6):763. doi: 10.3390/antibiotics11060763 (PMC9219656; doi:10.3390/antibiotics11060763)
Supplement: Supplementary file 1 [file antibiotics-11-00763-s001.zip › antibiotics-1750141-supplementary.pdf]

Supplementary Materials

Table S1. Characteristics in each secondary medical area.

Classification *1	Secondary Medical Area	Populations *2	Number of Hospitals *3	Number of Clinics *3
**Mie Prefecture**				
Big city	Hokusei	846,691	41	622
Small city-1	Chusei-iga	443,419	28	409
Small city-2	Nansei-shima	442,695	19	412
Small city-3	Higashikishuu	67,951	5	76
**Hiroshima prefecture**				
Big city-1	Hiroshima	1,366,272	95	1355
Big city-2	Fukuyama/Fuchu	513,758	46	383
Small city-1	Osan	242,054	23	200
Small city-2	Kure	240,046	30	241
Small city-3	Hiroshima Central	221,244	20	172
Small city-4	Hiroshima Nishi	143,661	13	125
Small city-5	Bihoku	85,442	10	87

*1: A population of 500,000 or more was defined as a "Big city," while a population of less than 500,000 was defined as a "Small city".; *2: Data from 2021 was used for the population [18].; *3:The number of healthcare facilities used data from 2019 [18].

Table S2. Changes of previously reported AMU.

	**Mie**				**Hiroshima**			
	**< 15 Years**	**15-64 Years**	**> 64 Years**	**All Ages**	**< 15 Years**	**15-64 Years**	**> 64 Years**	**All Ages**
**Third-generation cephalosporins**	↑	↑	↑	↑	↑	↑	↑	↑
**Quinolones**	↑	↑	↑	↑	↑	↑	↓	↑
**Macrolides**	↑	↑	↑	↑	↓	↑	↓	↑
**Total**	↑	↑	↑	↑	↑	↑	↑	↑

The increase is indicated by "↑" and the decrease by "↓". The gray areas show the antibiotics and age groups with decreased AMU. AMU: Antimicrobial use.

Table S3. Changes in oral antibiotic use stratified by age group and secondary medical area in the Mie and the Hiroshima prefectures from 2013 to 2016.

	Inpatients				Outpatients			
**Third-generation cephalosporins**	**< 15 Years**	**15-64 Years**	**> 64 Years**	**All Ages**	**< 15 Years**	**15-64 Years**	**> 64 Years**	**All Ages**
				**Mie Prefecture**				
Big city	0.0023	-0.00068	-0.0015	-0.0015	0.016	0.037	0.084	0.016
Small city-1	-0.0022	-0.0028	0.0075	-0.0019	-0.31	0.0029	0.081	-0.049
Small city-2	-0.0013	-0.0061	-0.012	-0.0090	0.047	0.034	0.096	0.012
Small city-3	0.00	-0.00087	-0.012	-0.0068	0.24	0.0011	0.10	0.014
**Quinolones**								
Big city	0.00045	0.00032	0.0061	0.00067	0.090	0.029	0.077	0.032
Small city-1	0.0025	0.00030	0.0088	0.00050	0.027	0.026	0.072	0.015
Small city-2	0.00014	0.00029	0.0073	-0.00013	0.090	0.033	0.095	0.030
Small city-3	0.00	-0.0019	0.012	0.00079	0.10	0.017	0.11	0.032
**Macrolides**								
Big city	0.0045	0.00018	0.0011	0.00071	0.029	0.056	0.098	0.041
Small city-1	-0.00025	-0.0011	-0.0025	-0.0020	0.027	0.028	0.063	0.0080
Small city-2	-0.0045	-0.00025	0.0023	-0.00077	-0.11	0.053	0.12	0.017
Small city-3	0.0051	-0.0014	-0.0062	-0.0032	-0.068	0.015	0.045	-0.012
**Total**								
Big city	0.012	0.00081	0.017	0.0029	0.43	0.16	0.32	0.16
Small city-1	0.0060	-0.0012	0.027	0.00081	0.12	0.081	0.27	0.039
Small city-2	-0.0017	-0.00031	0.030	-0.000074	0.034	0.18	0.41	0.11
Small city-3	0.0051	-0.0047	0.0072	-0.0067	0.43	0.058	0.32	0.067
				**Hiroshima Prefecture**				
Big city-1	-0.0052	-0.0033	-0.018	-0.0082	-0.0070	0.037	0.10	0.017
Big city-2	-0.0059	-0.00048	0.0012	-0.0026	0.079	0.0026	0.094	0.0038
Small city-1	-0.012	-0.0054	-0.00017	-0.0083	0.00096	0.030	0.099	0.0017
Small city-2	-0.0032	-0.010	-0.018	-0.015	-0.19	-0.0047	0.10	-0.032
Small city-3	-0.0072	-0.000094	-0.0038	-0.0035	0.18	0.10	0.12	0.086
Small city-4	-0.020	-0.0063	-0.0086	-0.010	0.00049	0.031	0.067	0.0045
Small city-5	-0.010	0.0027	0.021	-0.00010	-0.32	0.041	0.16	-0.016
**Quinolones**								
Big city-1	0.000031	-0.0014	0.0045	-0.0011	0.10	0.023	0.061	0.022
Big city-2	0.00084	-0.0018	0.00060	-0.0034	0.094	0.017	0.057	0.0091
Small city-1	-0.0036	-0.0043	-0.014	-0.011	0.044	-0.0031	0.065	-0.010
Small city-2	0.0035	-0.0022	-0.013	-0.0079	0.019	0.0089	0.021	-0.016
Small city-3	0.00010	-0.0026	-0.014	-0.0067	-0.020	-0.026	0.026	-0.036
Small city-4	-0.0015	-0.0034	-0.0065	-0.0055	0.17	0.086	0.15	0.080
Small city-5	0.0010	0.0022	0.00090	-0.0035	-0.10	-0.10	0.056	-0.10
**Macrolides**								

Big city-1	0.00044	0.00034	0.0014	0.00025	-0.067	0.056	0.082	0.023
Big city-2	0.0069	0.00040	-0.00054	0.00029	-0.13	0.033	0.068	-0.0060
Small city-1	0.0043	0.00065	-0.00015	-0.00047	-0.11	0.048	0.086	0.0064
Small city-2	0.0019	0.00034	0.0032	0.00078	0.018	0.031	0.076	0.012
Small city-3	-0.0021	-0.0017	-0.0026	-0.0024	-0.037	-0.024	0.0083	-0.040
Small city-4	-0.0058	0.00019	-0.0040	-0.0022	0.024	0.052	0.064	0.028
Small city-5	-0.0035	-0.0010	0.0029	-0.0017	-0.068	0.081	0.11	0.026
**Total**								
Big city-1	-0.0018	-0.0017	0.0063	-0.0041	0.35	0.16	0.31	0.14
Big city-2	0.0053	-0.00032	0.012	-0.0033	0.21	0.096	0.31	0.064
Small city-1	-0.0072	-0.0063	0.014	-0.012	0.13	0.13	0.36	0.067
Small city-2	0.0043	-0.0091	0.012	-0.011	0.013	0.060	0.29	0.0068
Small city-3	-0.010	-0.0017	-0.0046	-0.0082	0.61	0.16	0.25	0.15
Small city-4	-0.020	-0.0057	-0.020	-0.016	0.97	0.20	0.30	0.22
Small city-5	-0.014	0.0057	0.023	-0.0081	-0.70	0.029	0.41	-0.10

The values show the ΔPID, which is the difference between the PIDs in 2013 and 2016. The gray areas show the regions and age groups with decreased AMU. A population of 500,000 or more was defined as a “Big city”, while a population of less than 500,000 was defined as a "Small city”. AMU: Antimicrobial use. PID: Patients/1000 inhabitants/day.

Table S4. The concordance rate of changes in AMU of the secondary medical areas in the Mie and the Hiroshima prefecture and previously reported AMUs.

	**Japan**				**Mie**			
	**<15 Years**	**15-64 Years**	**>64 Years**	**All Ages**	**< 15 Years**	**15-64 Years**	**> 64 Years**	**All Ages**
**Mie Prefecture**								
**Third-generation cephalosporins**	37.5%	50.0%	62.5%	37.5%	62.5%	50.0%	62.5%	37.5%
**Quinolones**	100.0%	87.5%	0.0%	87.5%	100.0%	87.5%	100.0%	87.5%
**Macrolides**	50.0%	62.5%	25.0%	50.0%	50.0%	62.5%	75.0%	50.0%
**Total**	87.5%	62.5%	100.0%	75.0%	87.5%	62.5%	100.0%	75.0%
**Hiroshima Prefecture**								
**Third-generation cephalosporins**	71.4%	50.0%	64.3%	35.7%	28.6%	50.0%	64.3%	35.7%
**Quinolones**	71.4%	35.7%	28.6%	21.4%	71.4%	35.7%	28.6%	21.4%
**Macrolides**	42.9%	78.6%	28.6%	57.1%	57.1%	78.6%	28.6%	57.1%
**Total**	57.1%	57.1%	85.7%	42.9%	57.1%	57.1%	85.7%	42.9%

The gray areas show a concordance rate of 50% or less. AMU: Antimicrobial use.

(a)

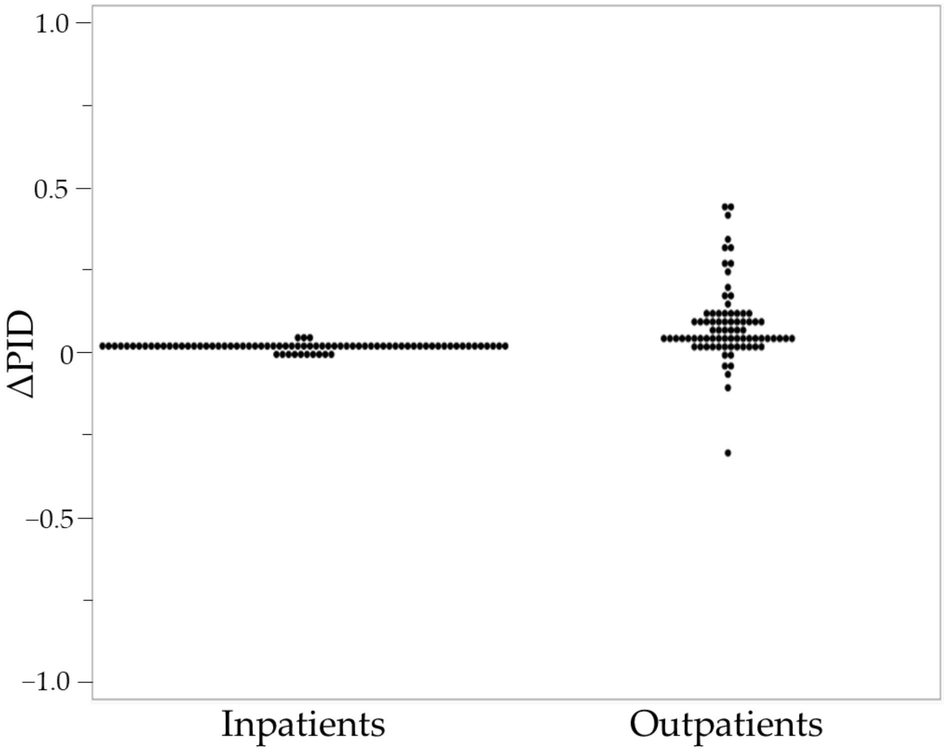

(b)

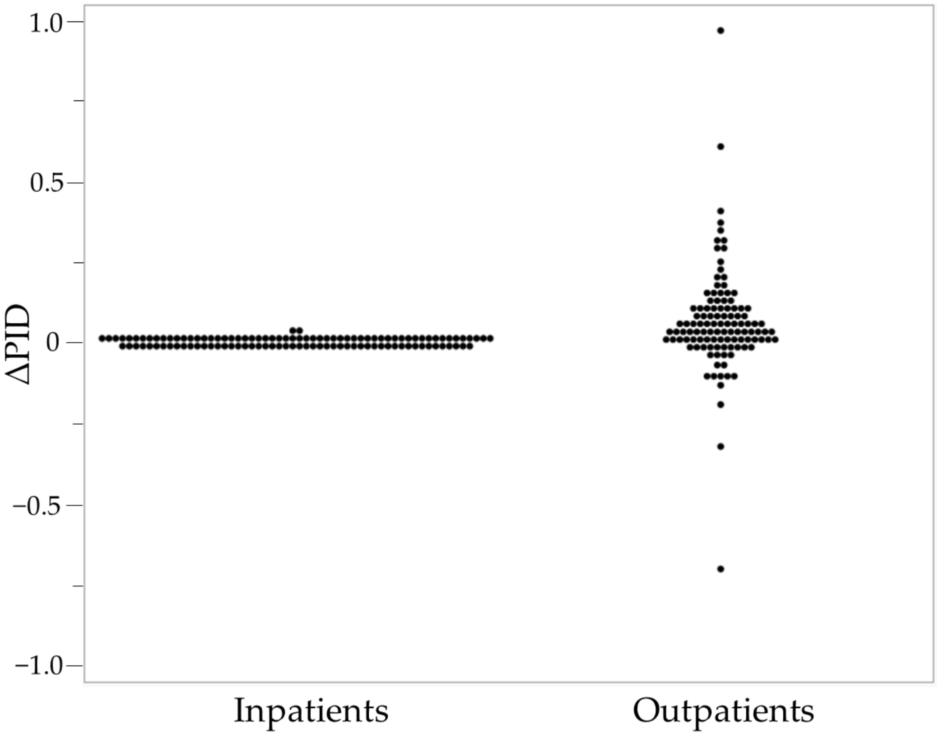

Figure S1. Bee-swarm plots of the ΔPID for inpatients and outpatients in two prefectures: Mie (a), Hiroshima (b). The left axis shows the ΔPID of each secondary medical area. The lower axis shows the number of inpatients and outpatients. PID: Patients/1000 inhabitants/day.
